# Maintenance of Cell Fate by the Polycomb Group Gene Sex Combs Extra Enables a Partial Epithelial Mesenchymal Transition in *Drosophila*

**DOI:** 10.1534/g3.120.401785

**Published:** 2020-10-13

**Authors:** Grace Jefferies, Jason Somers, Isabelle Lohrey, Vishal Chaturvedi, Jacob Calabria, Owen J. Marshall, Tony D. Southall, Robert Saint, Michael J. Murray

**Affiliations:** * School of BioSciences, University of Melbourne, Melbourne, VIC, Australia; † Menzies Institute for Medical Research, University of Tasmania, Hobart, TAS, Australia; ‡ Imperial College London, Sir Ernst Chain Building, South Kensington Campus, London, UK

**Keywords:** epithelial mesenchymal transition, *Drosophila*, Polycomb Group, Abdominal B, Sex combs extra, epigenetics, Targeted DamID, wing eversion

## Abstract

Epigenetic silencing by Polycomb group (PcG) complexes can promote epithelial-mesenchymal transition (EMT) and stemness and is associated with malignancy of solid cancers. Here we report a role for *Drosophila* PcG repression in a partial EMT event that occurs during wing disc eversion, an early event during metamorphosis. In a screen for genes required for eversion we identified the PcG genes *Sex combs extra* (*Sce*) and *Sex combs midleg (**Scm**)*. Depletion of *Sce* or *Scm* resulted in internalized wings and thoracic clefts, and loss of *Sce* inhibited the EMT of the peripodial epithelium and basement membrane breakdown, ex vivo. Targeted DamID (TaDa) using Dam-Pol II showed that *Sce* knockdown caused a genomic transcriptional response consistent with a shift toward a more stable epithelial fate. Surprisingly only 17 genes were significantly upregulated in *Sce*-depleted cells, including *Abd-B*, *abd-A*, *caudal*, and *nubbin*. Each of these loci were enriched for Dam-Pc binding. Of the four genes, only Abd-B was robustly upregulated in cells lacking *Sce* expression. RNAi knockdown of all four genes could partly suppress the *Sce* RNAi eversion phenotype, though *Abd-B* had the strongest effect. Our results suggest that in the absence of continued PcG repression peripodial cells express genes such as *Abd-B*, which promote epithelial state and thereby disrupt eversion. Our results emphasize the important role that PcG suppression can play in maintaining cell states required for morphogenetic events throughout development and suggest that PcG repression of Hox genes may affect epithelial traits that could contribute to metastasis.

Epithelial mesenchymal transitions (EMT) are a fundamental mechanism in development, homeostasis and pathologies such as cancer metastasis (Thiery *et al.* 2009). Since the genes that regulate EMT are highly conserved, studies in model organisms like the vinegar fly, *Drosophila melanogaster*, can play an important role in identifying and analyzing EMT factors important to human health. To find new EMT regulators in the fly we made use of a partial EMT event that occurs during imaginal wing disc eversion (Pastor-Pareja *et al.* 2004; Manhire-Heath *et al.* 2013; Murray 2015).

Eversion is an early event during metamorphosis whereby wing imaginal discs, and other imaginal discs, break through the larval epidermis and join up to create the new epidermis of the adult body. During eversion, peripodial epithelial (PE) cells exhibit classic hallmarks of EMT: they lose epithelial features, such as apico-basal polarity and adherens junctions, they express matrix metalloproteases that breakdown the basement membrane, and they become migratory, extending F-Actin rich protrusions. These cellular changes allow them to invade the overlying larval epidermis, creating perforations that coalesce and allow the wing discs to be externalized, and subsequently lead the epithelial migration that results in thorax closure. Failure of any these events can disrupt eversion leading to loss of thoracic tissue and midline clefts, and disruptions to the wings, including internalization, mis-positioning and reduction in size (Martín-Blanco *et al.* 2000; Pastor-Pareja *et al.* 2004; Ishimaru *et al.* 2004; Srivastava *et al.* 2007; Manhire-Heath *et al.* 2013).

To find EMT factors, we conducted an RNAi screen in which the *Ubx-GAL4* driver, which expresses strongly in peripodial cells, was used to knockdown genes during third-instar larval development, and adult flies (both eclosed and pharate), were scored for eversion defects (Golenkina *et al.* 2021). This screen identified Netrin-A (NetA) as a key regulator of the peripodial EMT (Manhire-Heath *et al.* 2013). NetA facilitates the breakdown of the adherens junctions of the peripodial epithelium (PE) via downregulation of its receptor Frazzled.

Here we present our analysis of another gene identified in this screen, the Polycomb Group (PcG) gene: *Sex combs extra (**Sce**)*. Sce is a *Drosophila* ortholog of vertebrate RING1, an E3 ubiquitin-ligase that monoubiquitinates H2A at K118 leading to chromatin compaction (Fritsch *et al.* 2003; Gorfinkiel *et al.* 2004). In *Drosophila*, PcG genes are well-known for their role in maintaining the patterns of Hox gene expression that are established during embryogenesis (Beuchle *et al.* 2001) but have not previously been associated with regulation of epithelial plasticity. In humans the PcG components EZH2 and Bmi1 have been linked with increased EMT and metastasis in cancer (Kleer *et al.* 2003; Wu and Yang 2011; Tong *et al.* 2012) as well as EMT during endometriosis (Zhang, Dong, *et al.* 2017). EZH2 forms a complex with Snail and HDAC1/HDAC2 to repress E-Cadherin expression (Cao *et al.* 2008; Tong *et al.* 2012), while Bmi1 cooperates with Twist to again silence E-Cadherin expression as well as the tumor suppressor p16INK4A (Yang *et al.* 2010; Wu and Yang 2011).

Here we show that loss of *Sce* results in a general failure of the wing disc to undergo the partial EMT of the PE, with effects on both the breakdown of zonula adherens (ZA) and basement membrane (BM). DamID transcriptional profiling revealed that *Sce* knockdown resulted in de-repression of the well-established PcG target genes *abd-A* and *Abd-B* along with a small group of other genes, which together comprise a strong epithelial signature. We found that Abd-B was upregulated in cells lacking *Sce* and RNAi knockdown of *Abd-B* was able to substantially repress the *Sce* RNAi phenotypes. Misregulation of Abd-B is clearly only partly responsible for the *Sce* phenotypes, however, as knockdown of other genes was also able to rescue to some extent, and ectopic expression of Abd-B, while having potent effects on epithelial morphology, did not, itself, recapitulate the *Sce.IR* phenotypes. Our results suggest that PcG activity in peripodial cells is required to keep them in a cell state that is competent to undergo the pEMT required for successful eversion. Loss of PcG repression causes a general shift in gene expression toward a more epithelial state, which inhibits eversion.

## Materials and Methods

### Drosophila stocks and husbandry

The following fly stocks were used in this study: *Ubx-GAL4* (Pallavi and Shashidhara 2003), *puc-GAL4* (Pastor-Pareja *et al.* 2004), *odd-GAL4* (Larsen *et al.* 2006), The following strains were obtained from the Bloomington Drosophila Stock Center at Indiana University: *Tre-GFP* (#59010), *UAS-Abd-B* (#913), *UAS-abd-A* (#912). All UAS-RNAi stocks were obtained either from the Vienna Drosophila RNAi Centre or the Bloomington Stock Centre. *Sce^KO^* (Gutiérrez *et al.* 2012) was a kind gift from J. Müller. Targeted DamID was carried out by crossing *Ubx-GAL4,GAL80^ts^* or *UAS-Sce.IR^V106328^;Ubx-GAL4,GAL80^ts^* flies to *UAS-mCherry-Dam-Pol II (attP2)* and *UAS-LT3-Dam(attP2)*, or *TaDaG-Dam (attP2)* and *TaDaG-Polycomb (attP2)* flies (Delandre *et al.* 2020). MARCM clones were created by crossing *hsFLP,UAS GFP;tub-GAL4, FRT82B tubP-GAL80* males to *w;FRT82B Sce^KO^* virgins and heat-shocking larvae at approximately early second instar for 30 min.

### Targeted DamID

The Targeted DamID protocol was as described (Marshall and Brand 2017), with minor alterations. For each replicate of each genotype, 30 wing discs were dissected from wandering third instar larvae in 1xPBS, pooled, excess PBS removed, and then frozen at -80° until required. Tissue was processed using a Qiagen DNeasy Kit. For the Dam-Pol II experiments, tissue from the freezer was thawed, 40ul of 500mM EDTA, 180ul of ATL buffer, and 20ul Proteinase K added, mixed gently and incubated for 56° overnight, cooled to RT and 20ul of RNAase (12.5ul/ul) added and incubated for 2 min 400ul of a 1:1 mix of Buffer AL and 100% ethanol was added and mixed gently, before processing the solution through the DNeasy kit spin columns. The genomic DNA was then digested overnight with DpnI, cleaned up with a Qiagen PCR Purification kit, and DamID Adapters blunt ligated with T4 ligase, digested again with DpnII, and then adapter-ligated fragments PCR amplified using DamID primers and Advantage PCR kit DNA polymerase (Clontech). Adapters were then removed with AlwI digestion, and final DNA fragments processed by the Melbourne Australian Genome Research Facility with a shotgun library prep protocol and 100bp single end reads generated on an Illumina HiSeq machine. For the Dam-Polycomb experiment, wing discs were prepared in the same way, though MyTaq polymerase (Bioline) was used for amplification, a TruSeq Nano Low throughput kit (Illumina) was used for library preparation and 86 base single-end reads were obtained on an Illumina MiSeq.

### damidseq_pipeline, genome visualization and statistical analysis

Sequencing data for Targeted DamID were mapped to release 6.03 of the Drosophila genome using damidseq_pipeline (Marshall and Brand 2015). Transcribed genes (defined by Pol II occupancy) were identified using a Perl script described in (Mundorf *et al.* 2019) based on one developed by (Southall *et al.* 2013) (available at https://github.com/tonysouthall/Dam-RNA_POLII_analysis). Drosophila genome annotation release 6.03 was used, with a 1% threshold. To compare data sets, log2 ratios were subtracted, in this case, producing 2 replicate comparison files (as 2 biological replicates were performed). These data were then analyzed as described above to identify genes with significantly different Pol II occupancy. Due to the presence of negative log2 ratios in DamID experiments, these genes were filtered to check that any significantly enriched genes were also bound by Pol II in the experiment of interest (numerator data set). A gene list was generated from the transcript data using the values from the associated transcript with the most significant FDR.

Replicate bedgraph files for each genotype were scaled by dividing each dataset by its standard deviation and averaged to create the profiles shown in Figures 2 and Fig. S3 which were visualized using pyGenomeTracks (Ramírez *et al.* 2018). Gene Ontology enrichment analysis was carried out using Flymine (Lyne *et al.* 2007).

For the Dam-Pc *vs.* Dam-Pol II analysis, log2 ratios were first scaled by the standard deviation and averaged, and then filtered to only include genes with significant occupancy in the Dam-Pc control, significant occupancy of Dam-Pol II in both genotypes, and with Dam-Pc occupancy >1 in control and below one in the Sce.IR discs.

### Immunohistochemistry and tissue culture

Wing disc dissection and culture, and immunostaining protocols were as previously described (Manhire-Heath *et al.* 2013). The following antibodies were used: guinea-pig anti-abd-A (used at 1:250) (Li-Kroeger *et al.* 2008); mouse-anti-Abd-B (Developmental Studies Hybridoma Bank (DSHB), 1A2E9, used 1:250); rabbit-anti-Caudal (a kind gift from Mark Biggin, 1:500); rat-anti-DE-Cadherin (DCAD2, DSHB, 1:250); rabbit-anti-Frazzled ((Kolodziej *et al.* 1996), 1:500); rabbit-anti-GFP (Life Technologies, 1:500); rabbit-anti-laminin β1 (Abcam, 1:250; rabbit-anti-Sex combs extra (a kind gift from M. Vidal, (Gorfinkiel *et al.* 2004), 1:500); rabbit-anti-Nubbin ((Terriente *et al.* 2008), 1:500). Rhodamine-Phalloidin was used at 1:100 (Cytoskeleton Inc.). Secondary antibodies (Jackson ImmunoResearch, 1:100) were all highly cross-absorbed varieties.

### Statistics

Fisher’s exact test (two-tailed) was used for comparison of proportions of categories in disc culture and adult eversion tests. All 95% confidence intervals were calculated using the Wilson score method with no continuity correction.

### Data availability

Reagents generated in this study are available on request. Figure S1 shows a validation of Sce knockdown. Figure S2 shows the peripodial driver expression patterns. Figure S3 shows that JNK activation and Fra expression are unaffected in Sce.IR discs. Figure S4 shows the Sce.IR derepression loci. Supplementary Data File1 shows gene lists showing Targeted DamID comparison of RNApol2 using the Ubx-GAL4 driver in third instar wing discs, with and without Sex Combs Extra RNAi. Transcriptome files generated in this study have been uploaded to the Gene Expression Omnibus (Edgar *et al.* 2002), Reference Series GSE153905. Supplemental material available at figshare: https://doi.org/10.25387/g3.12606437.

## Results

### Polycomb group gene expression in the peripodial epithelium is required for wing disc eversion

**Figure 1:**
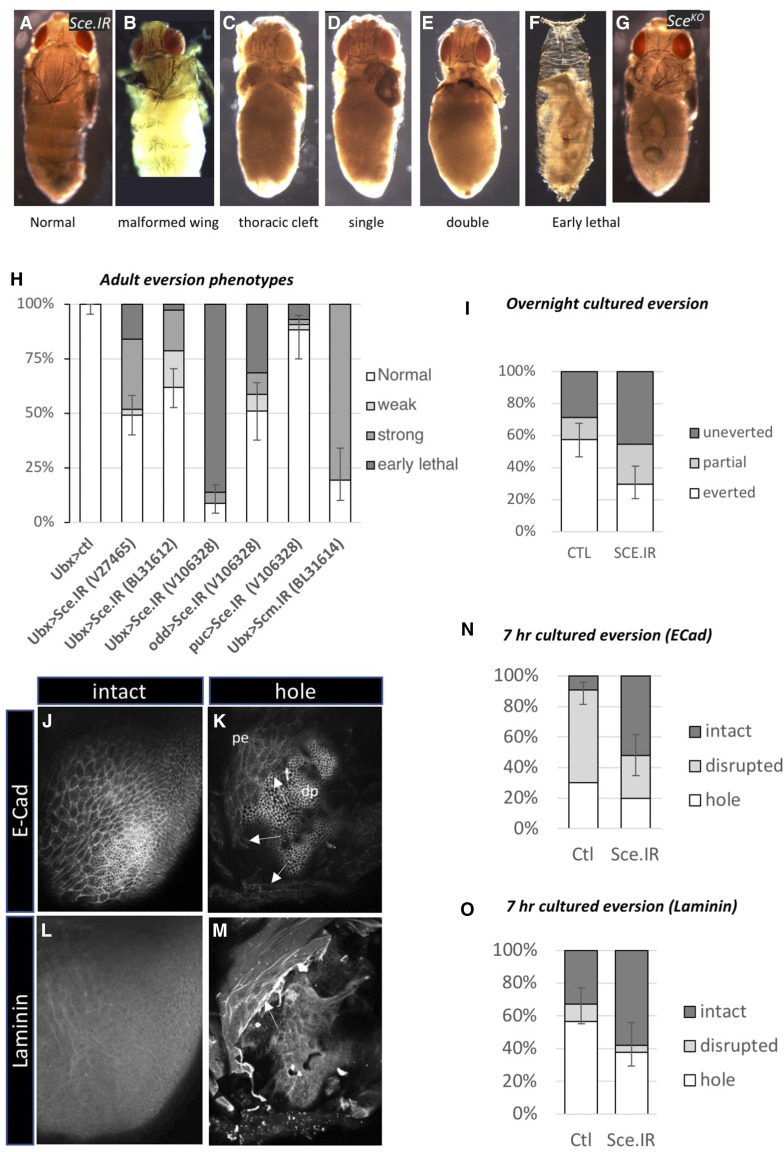
Sce expression in peripodial cells is required for wing eversion. (A-F) *Ubx* > *Sce.IR^V10638^* pupae showing increasingly severe categories of adult eversion failure. (G) A *Sce^KO^* MARCM pupa showing a thoracic cleft. (H) Quantification of adult eversion phenotypes. Knockdown of *Sce* using three different UAS RNAi lines, and three different peripodial GAL4 drivers (*Ubx-GAL4*, *puc-GAL4*, *odd-GAL4*) induces eversion failure phenotypes. Weak phenotype = malformed wing; Strong phenotype = thoracic clefts, single, and double eversion failure (see Table 1). (I) Overnight eversion of cultured third instar imaginal wing discs is inhibited by *Ubx-GAL4* driven knockdown of *Sce* (Ctl, n = 80; Sce.IR, n = 77; proportion everted *P* = 0.0007) (J-M) Categories of partial EMT and BM breakdown in wing discs cultured for 7 hr. (J) A disc stained for ECad showing intact AJs. (K) A wing disc with a substantial hole in the PE (pe) (arrows), with the underlying disc proper epithelium (dp) showing through. (L) A disc showing no signs of BM breakdown. (M) A disc in which the BM has developed a substantial hole and is pulling away. (N-O) Quantification of 7 hr eversion results. (N) Knockdown of *Sce* significantly inhibits breakdown of AJs and formation of holes in the PE layer (Ctl, n = 66; Sce.IR, n = 50; proportion intact *P* < 0.0001). (O) Knockdown of *Sce* significantly inhibits BM breakdown (Ctl, n = 67; Sce.IR, n = 50; proportion intact *P* = 0.00845). Error bars = 95% confidence interval (Wilson score method).

To find genes required for the peripodial EMT the *Ubx-GAL4* driver was crossed to UAS-RNAi lines and pharate or eclosed adult flies screened for eversion defects. Phenotypes were categorized in increasing level of severity (Figure 1) as:

- malformed wing; the thorax is normal but one or both wings are affected in some way such as being smaller, mispositioned, or crumpled (Figure 1B).
- thoracic cleft: both wings everted but a gap remaining in the middle of the thorax (Figure 1C);
- single-eversion failure: one wing failed to evert, resulting in an adult lacking half a thorax (Figure 1D);
- double-eversion failure: neither wing everted and thoracic tissue missing (Figure 1E);
- early pupal lethal: adult structures such as wings, legs and head not discernible (Figure 1F).

As expected, knockdown of genes known to play a role in eversion such as components of the JNK (*fos*, *slpr*) and TGFβ pathways (*dpp**, **punt*, *Mad*) generated eversion phenotypes (data not shown) as did *NetA* and *NetB* as previously described (Manhire-Heath *et al.* 2013). Two other genes with highly penetrant, and phenotypically severe, eversion defects were the PcG genes, *Sex combs extra (**Sce**)* and *Sex combs midleg* (*Scm**)*. Knockdown of these genes had similarly strong effects. RNAi to *Sce* using *UAS-Sce.IR^B31612^* resulted in a high proportion of single and double eversion failure (18.6%, n = 113) and crumpled wings (16.8%) (Table 1; Figure 1H). Similarly, knockdown of *Scm* with *UAS-Scm.IR^B31614^* produced high levels of single and double eversion failure (80.5%, n = 41; Table 1; Figure 1). For further analysis we focused our attention on *Sce*.

**Table 1:** Knockdown of the Polycomb Group genes, *Sce* and *Scm*, inhibits wing disc eversion

Genotype	Normal %	Weak %	Strong %	Early lethal %	n-val	p-val
+/+; Ubx-GAL4,GAL80^ts^/+	100.0	0.0	0.0	0.0	79	
Sce.IR^V27465^/+; Ubx-GAL4,GAL80^ts^/+	49.1	2.7	32.1	16.1	112	<0.0001
Sce.IR^BL31612^/+; Ubx-GAL4,GAL80^ts^/+	61.9	16.8	18.6	2.7	113	<0.0001
Sce.IR^V106328^/+; Ubx-GAL4,GAL80^ts^/+	8.9	0.0	5.1	86.1	79	<0.0001
Sce.IR^V106328^/odd-GAL4; +/+	51.0	7.8	9.8	31.4	51	<0.0001
Sce.IR^V106328^/+; puc-GAL4 /+	88.1	2.4	2.4	7.1	42	0.0043
Scm.IR^BL31614^/+; Ubx-GAL4,GAL80^ts^/+	19.5	0.0	80.5	0.0	41	<0.0001

p-values use two-tailed Fisher’s exact method on the proportion of normal adults.

To check for off-target effects, two other RNAi lines for *Sce* were tested: *UAS-Sce.IR^V106328^* and *UAS-Sce.IR^V27465^*. At 29° these also produced eversion defects, though in one case (*UAS-Sce.IR^V106328^*) the primary phenotype was early lethality (86.1%, n = 79; Table 1; Figure 1). However, subsequent tests using a temperature shift regime to restrict knockdown to a tighter developmental window, also produced a high proportion of double-eversion failures for this RNAi line (see below), suggesting that the early lethality was due to a stronger RNAi effect. Occasional eversion defects could also be generated by creating random *Sce^KO^* mutant clones using the MARCM technique (Lee and Luo 2001) (Figure 1G).

Immunostaining confirmed that Sce was expressed ubiquitously throughout the wing disc, including the peripodial epithelium, was predominantly nuclear, and appeared relatively constant between third instar and white prepupal stages (Fig. S1A, E). As expected there was a marked reduction of Sce levels in *Ubx > Sce.IR^V106328^* peripodial cells (Fig. S1C’’).

We next wished to see if *Sce* RNAi knockdown using other peripodial GAL4 drivers could also disrupt eversion. The PE has genetically distinct subdomains and different drivers express in different regions. The *Ubx-GAL4* driver has a broad expression domain throughout the central area of the PE but posterior to the anterior/posterior border, while the *odd-GAL4* driver expresses in the medial anterior cells, and the *puc-GAL4* driver, a reporter for JNK-activation, expresses strongly in peripodial cells nearest the stalk region (Pastor-Pareja *et al.* 2004; Tripura *et al.* 2011; Aldaz *et al.* 2013) (Fig. S2). Knockdown of *Sce* with both *odd-GAL4* and *puc-GAL4* produced eversion failures though the penetrance was less than for *Ubx-GAL4* (Table 1; Figure 1H).

Note that although *Ubx* is part of the bithorax complex along with *abd-A* and *Abd-B*, and that region is known to be regulated by PcG repression, our TaDa expression profiling showed that the *Ubx* locus was not affected by loss of *Sce* (see below) making it unlikely the *Ubx-GAL4* driver was itself being affected by loss of PcG repression.

Taken together these results show that *Sce* is required for eversion and suggest that target genes of PcG repression must remain repressed for successful eversion to occur.

### Sce RNAi affects the partial EMT of the wing discs

Since eversion is a complex multi-step process it can be affected at several stages: the initial apposition of the wing disc to the body wall, the degradation of the BM, the pEMT of the PE, the invasion of the epidermis, or the subsequent epithelial migration (Pastor-Pareja *et al.* 2004). Previously, we and others have found that the first steps of eversion, the pEMT and BM breakdown, can occur when discs are cultured in the presence of ecdysone (Milner 1977; Aldaz *et al.* 2010; Manhire-Heath *et al.* 2013). This provides an opportunity to determine if eversion failures are due to those early events, or later stages of the process. At 29°, eversion typically begins after 6-7 hr of culturing and is complete by 9-10 hr. To obtain an overall readout of eversion success we cultured discs for >16 hr, a period long enough to ensure complete eversion. Under these conditions we have found discs fall into three categories (Golenkina *et al.* 2021):

- successfully everted. discs that have flattened, wing-like morphologies and the PE forms a disorganised clump;
- partially everted. discs show evidence of breakdown of the PE but have not flattened out,
- uneverted. discs show no evidence of PE and BM breakdown although the DP may have undergone some bending.

When *Ubx > Sce.IR ^V106328^* discs (hereafter *Sce.IR* discs) were cultured overnight there was a significant change in eversion outcomes. The proportion of discs that were uneverted increased from 28.8% (n = 80) to 45.45% (n = 77) (*P* = 0.0007), while successful eversion fell by half, from 57.5 to 29.87% (Figure 1I).

Next, we looked at discs after 7 hr of culturing, which, at 29°, is a time when most discs are initiating epithelial dissociation by dismantling their AJs and are breaking down their BM. Discs were fixed and immunostained for E-Cadherin, Rhodamine-Phalloidin, and anti-Laminin to label AJs, F-Actin and BMs, respectively (Figure 1J-M). The 7hr results were consistent with overnight eversion. In control discs only 9.1% (n = 66) of discs showed an intact AJs compared to 52% (n = 50) in Sce.IR discs (*P* = 0.0001) – the remaining discs showing either a loss of AJs or small to large perforations in the PE (Figure 1N). Similarly, the proportion of discs with an intact BM was doubled from 32.8% of control discs to 58% of Sce.IR discs (*P* = 0.0085) (Figure 1O). Thus, there was overall inhibition of these processes in *Sce.IR* discs but no other obvious qualitative differences were detected.

Next, we tested whether two other key events in wing eversion were affected by loss of *Sce*: activation of the JNK pathway (Martín-Blanco *et al.* 2000; Pastor-Pareja *et al.* 2004; Srivastava *et al.* 2007), and downregulation of the Netrin receptor Frazzled (Manhire-Heath *et al.* 2013). However, expression of the JNK reporter Tre-RFP and Frazzled appeared normal (Fig. S3) suggesting that whatever genes were being misregulated, they were not involved in these pathways.

### Targeted DamID identifies de-repression of a small set of genes

To find which genes were affected we used Targeted DAMID (TaDa) with Dam-Pol II (Southall *et al.* 2013) to examine the change in transcriptional profile when *Sce* was knocked down. UAS-mCherry-Dam-Pol II and UAS-mCherry-Dam were expressed in control and *Sce.IR* discs and the ratios between Dam-Pol II and Dam profiles calculated (see Materials and Methods). Reproducibility between replicates was good with pair-wise Pearson correlation coefficients for GATC values over the genome between replicates ranging from 0.52-0.7 for control discs and 0.59-0.68 for *Sce.IR* discs.

**Figure 2:**
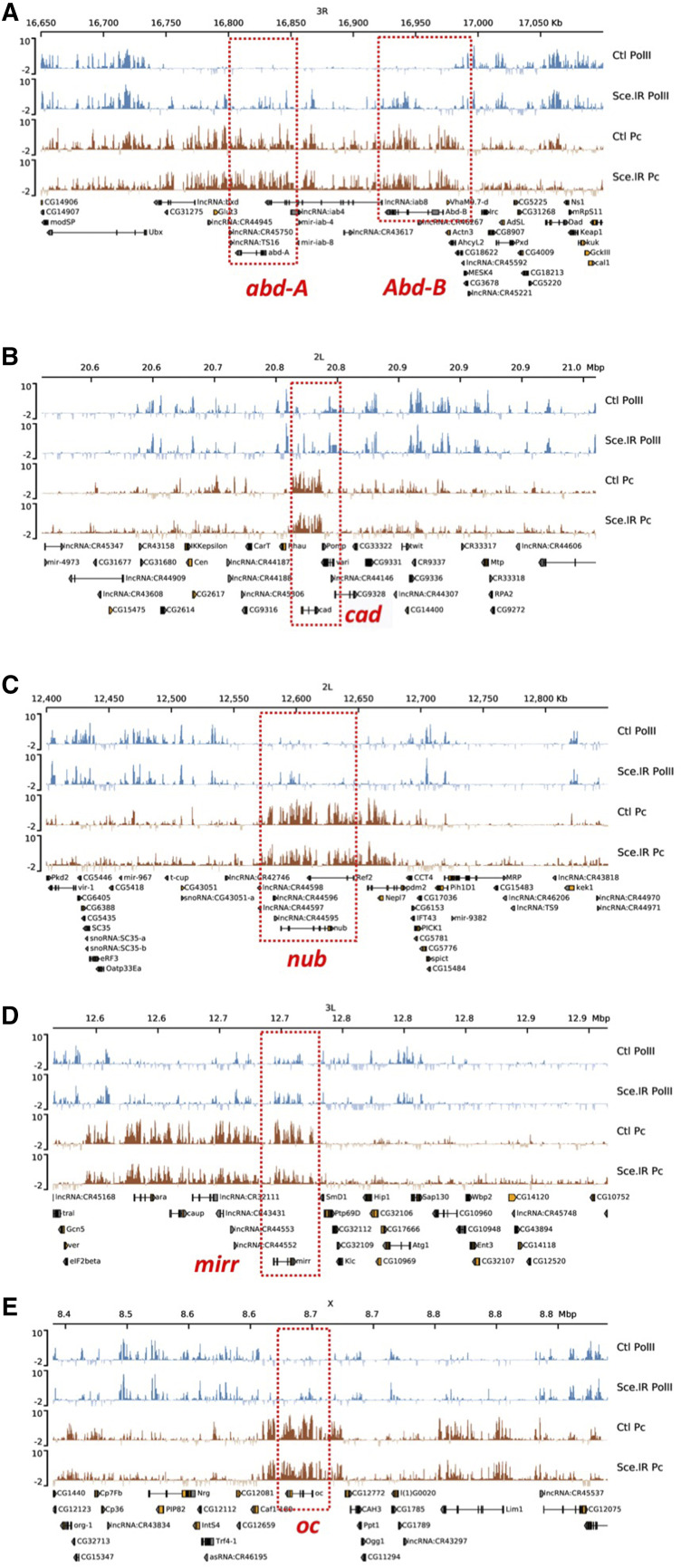
*Sce.IR* derepression loci are also Dam-Pc binding sites. (A-E) Genome browser views of major de-repression loci. Traces show fold-change of Dam-Pol II fusion over Dam alone, and Dam-Pc fusion over Dam alone for control discs and *Ubx > Sce.IR* discs. The bithorax region encompassing *abd-A* and *Abd-B* (A) is markedly derepressed in *Sce.IR* discs. These regions also show clear binding of Dam-Pc indicating that they are regions of PcG repression. Note that *Ubx* expression is unaffected by loss of *Sce*. Similarly, loci for *caudal* (B), *nubbin* (C), *mirror* (D) and *ocelliless* (E) show varying degrees of increased Dam-Pol II binding but are all clearly regions of Dam-Pc binding.

We determined the list of genes that were significantly expressed in both genotypes (FDR < 0.01; see Materials and Methods) and those whose expression was significantly increased or decreased in *Sce.IR* discs compared to control discs. 17 genes were significantly increased in *Sce.IR* discs (hereafter “de-repressed”) (Table 2; Figure 2; Fig. S4; Supp. File 1). This list included the well-known PcG targets *abd-A* and *Abd-B*. 110 genes showed a significant reduction in expression, with a fold-change of >1.3 (Supp. File 1).

**Table 2:** Genes significantly de-repressed in *Sce.IR* wing discs

Symbol	Name	Ratio*^a^*	FDR	Molecular function	Biological roles	GO-terms (biological function)
Abd-B	Abdominal-B	0.43916667	1.67E-19	Hox transcription factor	Bithorax complex Hox gene controlling posterior abdominal segments; external genitalia and gonads, and post-mating-response	epithelium development, anatomical structure morphogenesis
oc	ocelliless	0.17026667	2.66E-07	Paired-like homeobox transcription factor	regulator of rhodopsin expression and axonal targeting in the retina	epithelium development, anatomical structure morphogenesis
abd-A	abdominal-A	0.11543333	3.91E-07	Hox transcription factor	bithorax complex Hox gene controlling identity of embryonic segments	epithelium development, anatomical structure morphogenesis
mirr	mirror	0.1755	8.49E-06	iroquois homeobox transcription factor	dorso-ventral axis; eye formation; embryonic segmentation; PNS development	epithelium development, anatomical structure morphogenesis
nub	nubbin	0.08573333	1.61E-05	POU/homeodomain transcription factor	wing formation; midgut stem cell proliferation and enterocyte differentiation	anatomical structure morphogenesis
cad	caudal	0.17613333	4.41E-05	Hox-like homeobox transcription factor	anterior/posterior patterning, organ morphogenesis innate immune system	epithelium development, anatomical structure morphogenesis
Inx2	Innexin 2	0.21316667	0.00027954	gap junction protein	epithelial organization and polarity of epidermis, regulation of organ size and stem cell behavior	epithelium development, anatomical structure morphogenesis
CAH2	Carbonic anhydrase 2	0.35613333	0.00038658	Carbonic anhydrase	Catalyze the CO2 hydration reaction	
tup	tailup	0.06526667	0.0015028	LIM homeobox transcription factor	neuronal sub-type identity, including motor, serotonergic and dopaminergic neuron identity. It regulates germ band retraction, dorsal closure, muscle and heart development	epithelium development, anatomical structure morphogenesis
Sp1	Sp1	0.04683333	0.00190136	Sp-family of Cys2His2-type zinc finger transcription factors	ventral thoracic appendage specification; leg growth; type-II neuroblast development	epithelium development, anatomical structure morphogenesis
Pdk	Pdk	0.13876667	0.00347056	Pyruvate dehydrogenase kinase	Pyruvate dehydrogenase kinase	
Robo2	Roundabout-2	0.11006667	0.00481979	Robo-family cell surface receptor	Axon guidance receptor	epithelium development, anatomical structure morphogenesis
CG3777	CG3777	0.08913333	0.00529072	unknown	unknown	
pim	pimples	0.5068	0.00719975	Securin	Inhibits Separase	epithelium development, anatomical structure morphogenesis
CG3262	CG3262	0.39193333	0.00728925	unknown; interpro domain [Flagellum site-determining protein YlxH/ Fe-S cluster assembling factor NBP35]	unknown	
Psc	Posterior sex combs	0.09333333	0.00911746	Component of PRC1 complex	PcG epigenetic repression	Anatomical structure morphogenesis .
CG34293	CG34293-RA	0.61326667	0.00994297	Unknown; Interpro domain [Small subunit of serine palmitoyltransferase-like]	CG34293-RA	

a Values are for transcript isoform with highest ratio - see Supplementary Data File 1.

We performed Gene Ontology enrichment analysis on the lists of significantly changed genes, and on significantly expressed genes in the two genotypes (see Materials and Methods). For the 17 de-repressed genes, the most significant terms for biological function are “epithelium development” (10/17 genes; *P* = 0.001178; Holm-Bonferroni correction used for all enrichment analysis; Table 2) and “anatomical structure morphogenesis” (12/17 genes; *P* = 4.3e-4). There is also significant enrichment of genes with molecular function of transcription factors (8/17 genes; *P* = 4.37e-4), seven of which contain homeodomains. In contrast, for genes whose expression significantly decreased in *Sce.IR* discs there is no GO Term enrichment in any category.

Similar results were obtained when analysis was expanded to the entire set of significantly expressed genes in the two genotypes. The most strongly enriched biological function in *Sce.IR* discs is “epithelium development” (333/2045 genes; *P* = 1.84e-45), whereas for control discs it is “cellular-metabolic-process” (917/1898 genes; *P* = 2.8e-11). In *Sce.IR* discs there is also an enrichment of “cellular component” for cell junction proteins (62 genes; *P* = 2.0e-11) and of “molecular function” for actin binding (51 genes; *P* = 3.1e-7) consistent with cellular changes impacting upon pEMT processes.

Since direct targets of the PcG complexes would be expected to have increased expression we focused our attention on the 17 de-repressed genes. Changes in expression levels for these genes, averaged across the whole gene locus were relatively modest, ranging from 0.61 to 0.047 log_2_ (*i.e.*, fold-change of 1.5 to 1.03) averaged over the gene locus.

To confirm that these genes corresponded to regions of PcG repression we again used TaDa to examine the binding profile of the PcG component, Polycomb using UAS-myr-GFP-Dam-Polycomb and a UAS-myr-GFP-Dam control (Materials and Methods). The Dam-Pc ratio profile exhibited the expected genomic patterns of Polycomb binding for known PcG target areas, such as the *engrailed**/**invected* and the bithorax regions (Tolhuis *et al.* 2006) indicating that the method had worked. For each of the 17 genes we then calculated the average level of Pc-binding in control discs (Fig. S4B). The genes with the most significant fold-change in *Sce.IR* discs *vs.* controls (FDR < 1e-4) (Figure 2) also tended to have higher levels of Pc-binding (Fig. S4). We also examined the Dam-Pc profile in *Sce.IR* discs but found the pattern of binding largely unchanged from control discs though the average ratio levels across the genome were reduced (Figure 2; Fig. S4; and data not shown).

Thus, the loss of *Sce* has resulted in increased expression of a small number of genes in PcG-repression regions, and this is accompanied by a genome-wide change in genes from those associated with cellular metabolism to those involved in epithelial development, consistent with an inhibition of the PE pEMT.

### Abd-B is upregulated in the peripodial epithelium of Sce.IR discs and required for eversion failure

Based on the expression profiles of the de-repressed genes, we conducted further tests on four of the genes that had a distinct change in expression profile and higher levels of Pc-binding: *abd-A*, *Abd-B*, *cad* and *nub*.

**Figure 3:**
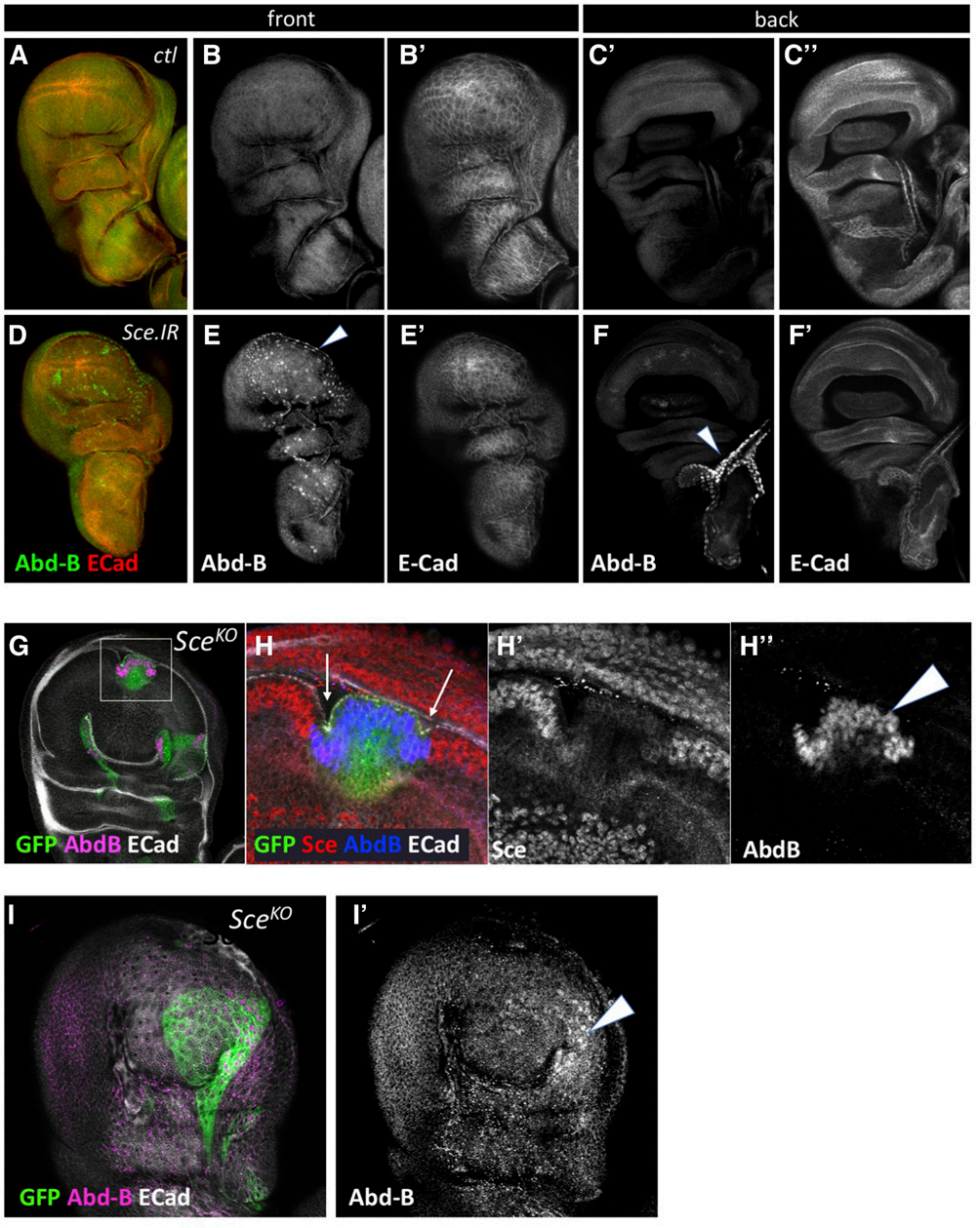
Sce represses Abd-B. (A-F) Third instar wing discs stained for Abd-B and E-Cadh. In control discs (A-C) no expression of Abd-B is detected. In *Sce.IR* discs, Abd-B is expressed in nuclei throughout the PE (E, arrowhead), and the tracheal branch on the side of the discs, opposite to the PE (F, arrowhead). (G-I) MARCM clones of *Sce* show clear upregulation of Abd-B in both the disc proper (H’’, arrowhead; I’, arrowhead) and PE (I’, arrowhead). Sce expression is clearly lost from MARCM clones (H’). MARCM clones show distinct morphological changes with invaginations at the boundary with surrounding heterozygous cells (H, arrows), and a smoother, more rounded profile in the PE (I, green).

We first used immunostaining to determine if any of the four genes showed significant upregulation in the PE of *Sce.IR* discs. Of the four genes, only Abd-B showed a clear change in expression in PE cells with nuclear staining apparent in the *Sce.IR* discs but not in control discs (Figure 3A-F). We further confirmed that loss of *Sce* was responsible for Abd-B upregulation by examining MARCM clones for the null allele *Sce^KO^*. Clones in both the PE and DP showed clear upregulation of Abd-B (Figure 3G-I). In addition, there was a morphological change in both PE and DP clones in that they showed a “segregation-phenotype” whereby they became more rounded and developed furrowing/invagination at the borders with wild type cells as previously reported for several PcG genes (Beuchle *et al.* 2001; Fritsch *et al.* 2003; Gandille *et al.* 2010; Curt *et al.* 2013).

**Figure 4:**
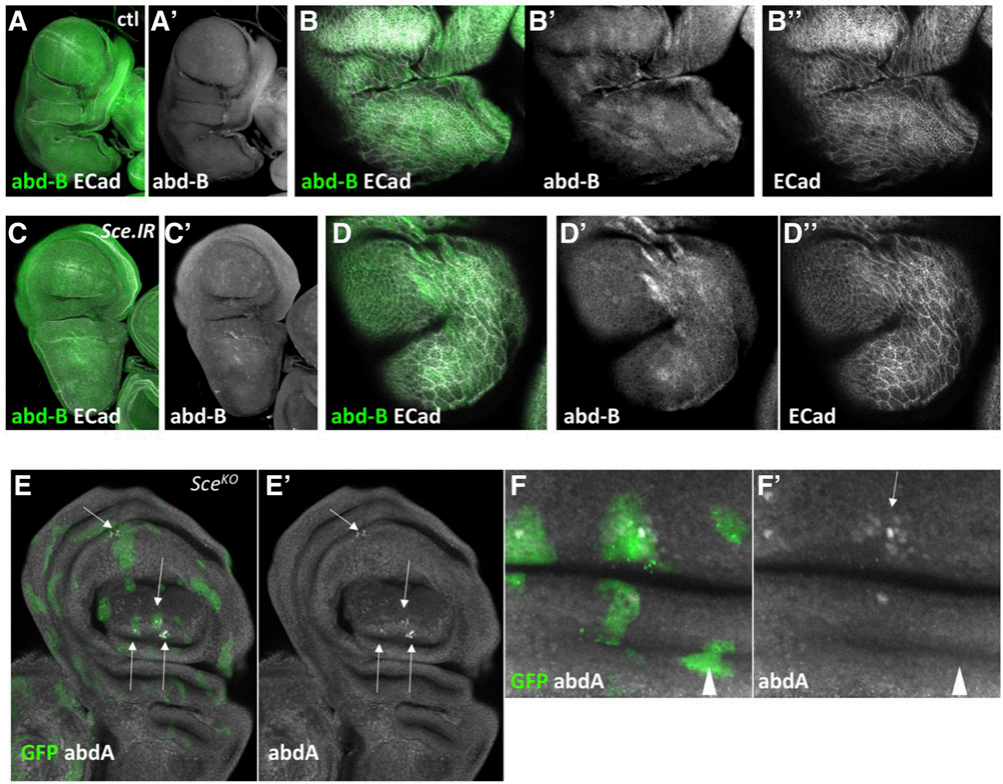
abd-A is partially repressed by Sce. (A-F) Third instar wing discs stained for abd-B and E-Cadh. In control discs (A-B) there is no nuclear expression of abd-B though some cytoplasmic staining in PE cells was apparent. (C-D) *Ubx > Sce.IR* discs, appeared the same, though the cytoplasmic staining appeared somewhat stronger. (E-F) In *Sce* MARCM discs there was clearly some nuclear expression of abd-A in some clones (E, E’, F, F’, arrows) though this was of varying strength within a clone (F’, arrow), and some clones showed no expression (F’, arrowhead).

Although no obvious change in abd-A, Nub or Cad expression/localization was seen in *Ubx > Sce.IR* PE cells, a subset of *Sce^KO^* MARCM clones also showed clear upregulation of abd-A, though the levels were variable (Figure 4). We speculate that while Abd-B is directly controlled by PcG complexes, abd-A faces more complex regulation and may be being suppressed by Abd-B and/or the non-coding RNA *mir-iab-8* which is also located in the de-repressed region between *abd-A* and *Abd-B*. In the case of Nub and Cad there was no nuclear expression though we cannot discount the possibility of a mild increase in cytoplasmic signal.

**Figure 5:**
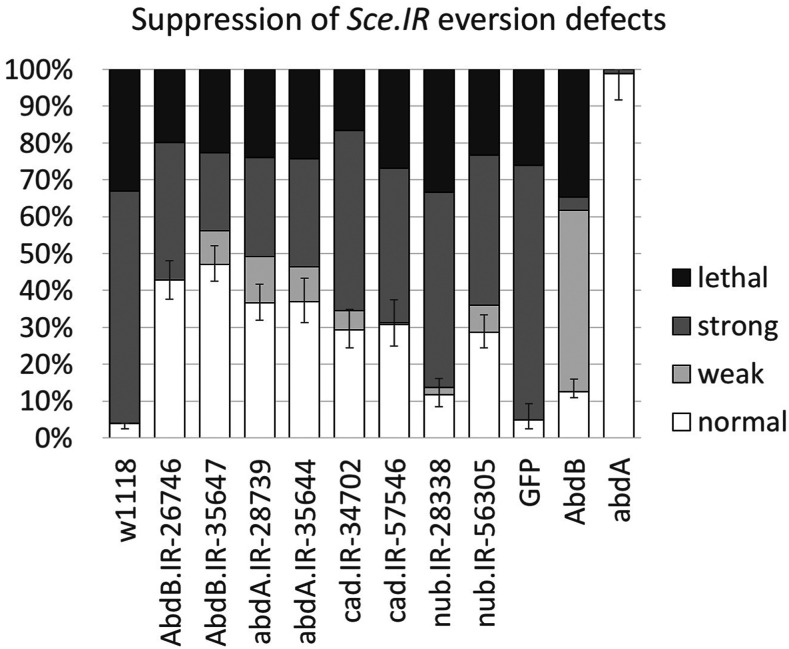
Knockdown of de-repressed loci substantially represses *Sce.IR* eversion phenotypes. Effects on adult eversion failure when *Ubx-GAL4* knockdown of *Sce* is accompanied by expression of the indicated UAS RNAi lines, or UAS-GFP control. Co-expression of GFP does not significantly decrease the rates of eversion failure in *Ubx > Sce.IR* discs, but co-expression of UAS RNAi lines for *Abd-B*, *abd-A*, *cad*, and *nub* all repress eversion failure. *Ubx-GAL4* expression of *Abd-Bm* produces a high proportion of weak phenotypes in which the thorax is normal, but wings are deformed or mispositioned (56%, n = 222). Expression of *abd-A* has no effect (n = 84). Error bars = 95% confidence interval (Wilson score method).

Next, we tested whether *RNAi* knockdown of any of the four genes could suppress the *Sce.IR* phenotypes. We utilized the *Sce.IR ^V106328^* RNAi line but used a temperature shift regime to restrict the period of GAL4 expression to third instar stages, thereby avoiding the excessive early pupal lethality. Two independent RNAi lines were used for each gene (Figure 5). Knockdown of any of the four genes was able to partly rescue the defects while co-expression of an arbitrary UAS construct, *UAS-GFP*, had no effect (normal progeny = 4.8%, n = 165, *P* = 0.65). Of the four genes loss of *Abd-B* had the strongest effect increasing the proportion of normal eversion from 4% in *Sce.IR* discs (n = 379) to 47.15% in *Sce.IR;Abd-B.IR* discs (n = 397, Figure 5; Table 3; *P* < 0.0001). The results suggest that the inhibition of eversion may not be due to any one of these genes, but rather to a genome-wide change in transcriptional profile toward an epithelial state. The other implication is that the maintenance of epithelial/BM integrity in *Sce.IR* discs is relatively unstable, since knockdown of any of the four PcG targets was enough to substantially restore successful eversion.

**Table 3:** Repression of *Sce.IR* eversion defects

Genotype	Normal %	Crumpled %	Cleft %	Single %	Double %	Lethal %	n-val	p-val
UAS-Sce.IR^V106328^/+;Ubx-GAL4, GAL80^ts^/+	4.0	0.0	4.0	7.4	51.7	33.0	379	
UAS-Sce.IR^V106328^/UAS-AbdB.IR^BL26746^;Ubx-GAL4,GAL80^ts^/+	42.8	0.0	28.3	4.9	4.0	19.9	346	<0.0001
UAS-Sce.IR^V106328^/UAS-AbdB.IR^BL35647^;Ubx-GAL4,GAL80^ts^/+	47.1	9.1	3.8	6.8	10.6	22.7	397	<0.0001
UAS-Sce.IR^V106328^/UAS-abdA.IR^BL28739^;Ubx-GAL4,GAL80^ts^/+	36.7	12.5	7.4	7.7	11.7	23.9	376	<0.0001
UAS-Sce.IR^V106328^/UAS-abdA.IR^BL35644^;Ubx-GAL4,GAL80^ts^/+	37.0	9.5	4.1	7.8	17.3	24.3	243	<0.0001
UAS-Sce.IR^V106328^/UAS-cad.IR^BL34702^;Ubx-GAL4,GAL80^ts^/+	29.3	5.2	13.1	9.3	26.6	16.6	290	<0.0001
UAS-Sce.IR^V106328^/UAS-cad.IR^BL57546^;Ubx-GAL4,GAL80^ts^/+	30.8	0.5	10.0	5.0	26.9	26.9	201	<0.0001
UAS-Sce.IR^V106328^/UAS-nub.IR^BL28338^;Ubx-GAL4,GAL80^ts^/+	11.8	1.8	5.9	5.9	41.2	33.5	272	0.0002
UAS-Sce.IR^V106328^/UAS-nub.IR^BL56305^;Ubx-GAL4,GAL80^ts^/+	28.7	7.2	15.5	7.5	17.8	23.3	387	<0.0001
UAS-Sce.IR^V106328^/UAS-GFP;Ubx-GAL4,GAL80^ts^/+	4.8	0.0	17.6	9.1	42.4	26.1	165	0.6464

p-values use two-tailed Fisher’s exact method on the proportion of normal adults.

**Figure 6:**
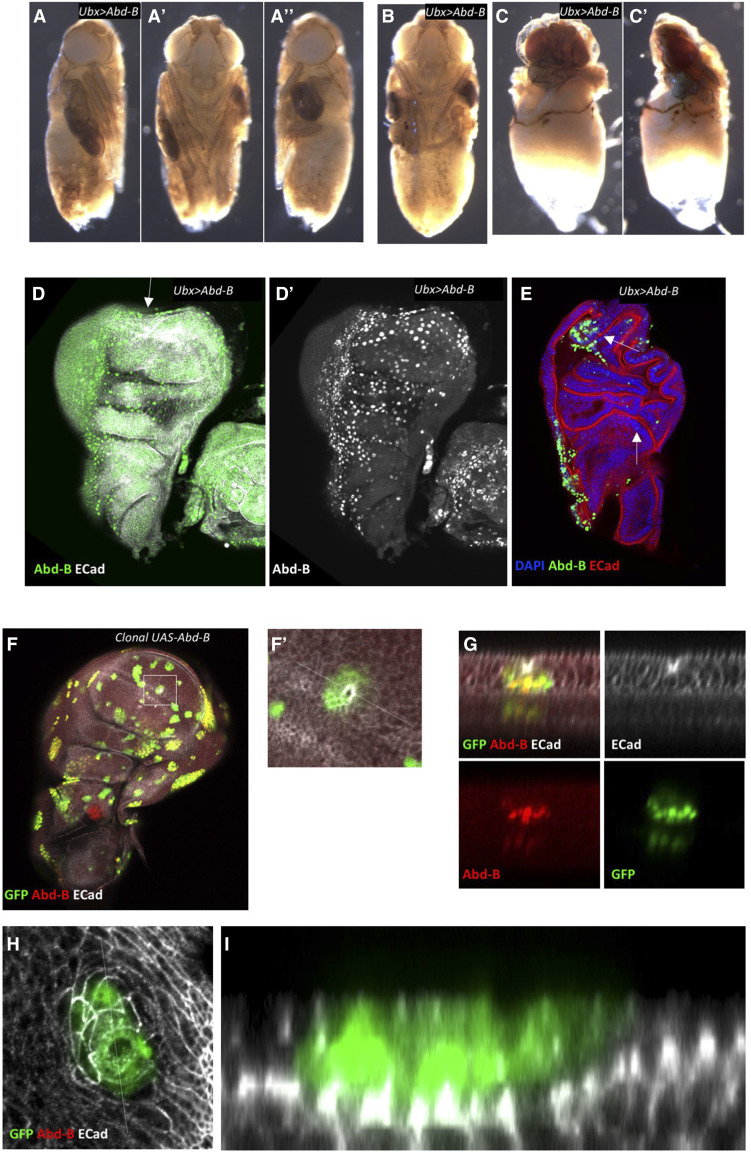
Abd-B affects epithelial cells and wing morphology. (A-C) Pharate adults showing eversion phenotypes. The primary phenotype was for one or more malformed and mispositioned wings (A’, A’’). (B) An adult with both wings affected, and legs malformed. (C) Adult with a more severe phenotype in which the thorax is disrupted and only one wing has everted, but is malformed. (D) Expression of *Abd-Bm* with the *Ubx-GAL4* driver induces strong expression of Abd-B throughout the PE (D’) but some regions of expression in the DP cells in the wing blade also robustly express Abd-B. Wing discs show a range of morphological disruption ranging from mild depressions (D, arrow) to more substantial folding (E, arrows). (F-I) Clonal expression of Abd-Bm creates regions of epithelial invagination. (F) A small clone of Abd-Bm expressing cells has folded inwards to produce a depression. (G) Cross-section of dotted line in F’. (H) A clone of Abd-Bm expressing cells in the PE creates a depression of the underlying DP epithelium. (I) Cross-section of dotted line in H. Error bars = 95% confidence interval (Wilson score method).

Finally, we tested whether over-expression of either of the two genes with strongest rescue, Abd-B and abd-A, could phenocopy loss of *Sce*. *Ubx-GAL4*-driven expression of Abd-B in the PE did not block eversion, though a high proportion of adults had reduced/misplaced wings (Figures 5, 6A-C). Clonal expression of Abd-B did, however, recapitulate the epithelial invagination/segregation phenotype, as has previously been described (Gandille *et al.* 2010) (Figure 6F-I). *Ubx-GAL4*-driver expression of *abd-A* had no effect, however clonal expression of abd-A also created invaginations suggesting that this phenotype is a conserved ability of Hox genes to regulate epithelial morphology (data not shown). Since sole expression of Abd-B was not able to recapitulate the *Sce.IR* phenotypes we conclude that while the epithelial morphology changes induced by Abd-B, and to a lesser extent, abd-A, may contribute to eversion failure, they are not sufficient.

Overall, our results imply that the eversion failure of *Sce.IR* discs is due to a genome-wide change in gene expression toward an epithelial state, and that Abd-B likely plays the major role in this change.

## Discussion

We have uncovered a new role for PcG repression during *Drosophila* development: maintenance of the state of peripodial cells such that they are able to undergo the partial EMT that allows eversion to proceed. Loss of *Sce* leads to de-repression of a small number of target genes and an overall shift in gene expression toward a cell-state associated with “epithelial development”, and hence eversion is impeded. Thus, PcG repression is not only crucial for maintaining segmental identity but also for maintaining cells in a state of readiness for the epithelial plasticity events that occur later during development and which are necessary for successful eversion.

Our TaDa analysis of Dam-Pol II binding identified a surprisingly small number of genes that were upregulated in *Sce.IR* discs. Only 17 genes had an FDR < 0.01 and two of these were the known PcG targets, *abd-A* and *Abd-B*. Using Dam-Pc we confirmed that, for most of these genes, their loci corresponded to Polycomb binding regions of the genome.

In contrast there were 110 genes that were significantly downregulated in *Sce.IR* discs but these showed no GO-term enrichments and did not include well-known *Drosophila* EMT regulators, such as Snail and Serpent. However, one gene that is linked to EMT in mammals, and was among the most significantly reduced genes, was the lipid raft protein Flotillin-1 (Flo1). In *Drosophila* Flo1 has been shown to regulate collagen turnover (Lee *et al.* 2014) which could well promote the eversion process. In mammals Flotillins are more strongly linked to EMT, where they promote endocytosis and turnover of both cell adhesion molecules and ECM proteins and promote cancer metastasis (Gauthier-Rouvière *et al.* 2020). Interestingly, the *Drosophila* paralog Flo2, is also upregulated during wound healing (Juarez *et al.* 2011), a cellular event with many parallels to thorax closure, including the involvement of Src42A and the JNK pathway. It will be of great interest, therefore, to explore the role of the two Flotillins in the eversion process.

We focused our attention on four of the genes with a clear change in Dam-Pol II profile and tested whether RNAi knockdown could repress the eversion defects of *Ubx > **Sce**.IR*. Surprisingly, we found that all had a significant effect on rescue, though the knockdown of *Abd-B* was the most significant. It is possible that co-expression of multiple UAS lines might result in a reduction in the strength of the *UAS-Sce.IR* phenotype, simply due to competition for GAL4. However, we found no effect of combined expression of UAS-GFP. We speculate that PE breakdown and the eversion process as a whole, are “threshold events” that tend to proceed to completion once begun - like a membrane tearing. In a genotype such as *Sce.IR*, where eversion is failing about half the time, the PE is presumably poised at that critical threshold – such that a small change in gene expression can have a large effect. Other dominant modifier tests we have conducted involving eversion have shown a similar sensitivity to genetic perturbation (data not shown). Although the expression of these genes was clearly important in blocking eversion, over-expression of *Abd-B* and *abd-A* on their own, was unable to recapitulate the eversion blockage, suggesting that it is the combined expression that produces a cell state necessary to inhibit the pEMT and BM breakdown.

Others have shown previously that loss of various PcG genes in wing discs results in ectopic expression of Ubx, Abd-B and Cad, and epithelial morphogenesis changes (Beuchle *et al.* 2001; Fritsch *et al.* 2003; Gandille *et al.* 2010; Curt *et al.* 2013). Interestingly, the results of this study for Sce and Scm clones (Beuchle *et al.* 2001) was that only Ubx and Abd-B were expressed in the time-window used. Our results agree with these in that we saw Abd-B upregulation, occasional abd-A upregulation but no Caudal. We did not look at Ubx protein expression in disc-proper cells. Abd-B expression was the clearest effect of loss of *Sce* and could induce clear morphological changes on epithelial cells. Abd-B plays a well characterized role in the formation of posterior spiracles in the embryo, and this also involves invagination of epithelial tissue. In that case a small downstream regulatory network has been established involving the four immediate target genes, *cut*, *spalt*, *upd1*, and *ems*, as well as *crumbs*, *Gef64C* and five cadherins (Lovegrove *et al.* 2006). None of these genes showed significant upregulation in *Sce.IR* discs, however, suggesting that there may exist other Abd-B targets that affect epithelial plasticity.

The importance of PcG repression of Abd-B has also been seen in the context of testes development and the closure of the tergites. PcG repression of Abd-B in cyst stem cells of the testes is critical for normal cell fate identity and self-renewal of the stem cells (Zhang, Pan, *et al.* 2017). Mutation of regulatory elements the Boundary Elements and Polycomb Response Elements can also cause increased and ectopic expression of Abd-B that results in dorsal closure defects in the adult abdominal epithelium (Singh and Mishra 2015).

While Abd-B was always derepressed in cells lacking Sce (*i.e.*, *Sce.IR* and *Sce^KO^* mutant cells) abd-A was intermittently and variably expressed. We speculate that this may be a manifestation of the posterior dominance rule, whereby expression of Abd-B expression can repress abd-A (Karch *et al.* 1990; Macías *et al.* 1990; Sánchez-Herrero 1991). It is also possible that *abd-A* is being regulated by the non-coding RNA *mir-iab-8* (Gummalla *et al.* 2012) since it is also located in the region of increased Dam-Pol II binding.

In conclusion, we have demonstrated a new role for PcG repression in maintaining cell competency for a developmental EMT event and shown that silencing of abd-A and Abd-B is crucial in this process. An important question now is what downstream targets of Abd-B and abd-A, and perhaps other TFs like Caudal and Nubbin, are inhibiting the pEMT and are these gene-regulatory interactions conserved in mammals. Based on the effects of EZH2 and Bmi1 on E-Cadherin, we expected increased expression at the *shg* locus in the Sce.IR discs, but this was not seen. Mammalian Hox genes control many processes involving epithelial plasticity such as cancer metastasis, wound healing and angiogenesis, but they can have both positive and negative effects (Abate-Shen 2002; Kachgal *et al.* 2012). For example, HOXB9 promotes differentiation and mesenchymal-epithelial transition, while inhibiting migration and invasion, in both colon adenocarcinoma (Zhan *et al.* 2014) and gastric carcinoma cells (Chang *et al.* 2015). Conversely, other studies have found the same gene is overexpressed in breast carcinoma cells and correlates with high tumor grade (Hayashida *et al.* 2010) and overexpression in colon cancer cells promotes metastasis and poor prognosis (Huang *et al.* 2014). Thus, understanding how epithelial plasticity is regulated by Hox genes is likely to be complex and context dependent, but remains an important future goal.
